# Measuring and modeling macrophage proliferation in a lab-on-CMOS capacitance sensing microsystem

**DOI:** 10.3389/fbioe.2023.1159004

**Published:** 2023-05-12

**Authors:** Kyle Smith, Ching-Yi Lin, Yann Gilpin, Elizabeth Wayne, Marc Dandin

**Affiliations:** ^1^ Department of Chemical Engineering, Pittsburgh, PA, United States; ^2^ Department of Electrical and Computer Engineering, Pittsburgh, PA, United States; ^3^ Department of Biomedical Engineering, Carnegie Mellon University, Pittsburgh, PA, United States

**Keywords:** CMOS, biosensors, capacitance, lab-on-chip, lab-on-CMOS, model, cell proliferation, macrophage-cell

## Abstract

We report on the use of a lab-on-CMOS biosensor platform for quantitatively tracking the proliferation of RAW 264.7 murine Balb/c macrophages. We show that macrophage proliferation correlates linearly with an average capacitance growth factor resulting from capacitance measurements at a plurality of electrodes dispersed in a sensing area of interest. We further show a temporal model that captures the cell number evolution in the area over long periods (e.g., 30 h). The model links the cell numbers and the average capacitance growth factor to describe the observed cell proliferation.

## 1 Introduction

Macrophages are innate immune cells that specialized inflammatory functions related to damage detection, pathogen recognition, clearance, and wound healing (Wynn et al., 2013). To perform these heterogeneous functions, macrophages undergo polarization whereby cells produce a specialized phenotype in response to environmental stimuli. Tissue-resident macrophages become polarized during the progression of infectious disease and chronic diseases like cancer, neurodegeneration, and autoimmunity (Williams et al., 2018; Ma et al., 2019; Reis-Sobreiro et al., 2021). As such, drug targeting for the purpose of re-polarizing macrophages is a major basic and translational research area.

Macrophage inflammatory functions can be detrimental and contribute to disease progression. For instance, atherosclerotic plaque progression and rupture can be fueled by macrophage-mediated inflammation (Tang et al., 2015). In another example, tumor-associated macrophages (TAMs) can produce chemokines that in turn promote tumor metastasis, immune escape, and lung metastasis in breast cancer (Zheng et al., 2023). In yet another example, the inflammatory responses involving infiltration and activation of liver macrophages can play a vital role in acute liver failure (Cai et al., 2023). Thus, in the management of the aforementioned diseases, attenuating local macrophage proliferation can be an appealing therapeutic target.

To facilitate *in vitro* studies of macrophage proliferation and to provide a tool for label-free and real-time mechanistic investigations, we present herein an electronic microsystem, specifically, a lab-on-CMOS platform, that achieves real time macrophage proliferation monitoring using a capacitance-sensing bioelectronic interface. A lab-on-CMOS device is a platform that integrates lab-on-a-chip technology with complementary metal-oxide semiconductor (CMOS) chips for biosensing (Roussel et al., 2006; Christen and Andreou, 2007; Sawan et al., 2010; Ghallab and Ismail, 2014; Yin et al., 2016; Mason and Wan, 2017; Li et al., 2018; Lin et al., 2022). The chips can feature circuits that are configured to transduce biophysical and biochemical events to the electrical domain and signal processing hardware for measurement, conditioning, and reporting. The biological species under test, in our case the macrophages and their liquid media, are applied directly on the chip’s surface where a plurality of sensors are disposed. Other parts of the chip are isolated from the cell media using a custom-designed package configured to preserve the chip’s electrical integrity while it is operating in a wet environment.

Our chip’s transduction mechanism is based on interfacial capacitance sensing (Forouhi et al., 2018; Forouhi et al., 2019). The principle of operation of interfacial capacitance sensing is like that of electrical cell-substrate impedance sensing (ECIS) (Susloparova et al., 2013; 2015; Hu et al., 2021). ECIS measures changes in impedance at a sensing electrode as a function of frequency and time, whereas interfacial capacitance biosensing tracks changes in capacitance at the sensing electrode as a function of time (Hedayatipour et al., 2019).

While both methods can yield the same information about a cell culture overlying a set of sensing electrodes, capacitance sensing circuit architectures are simpler to implement because the complex current sourcing and wide band frequency scanning circuitry typically used in ECIS schemes are not needed to sense changes in capacitance (Hedayatipour et al., 2019). Rather, simpler topologies can be employed to convert the sensed capacitance into a voltage, a current, a frequency, or a pulse width (Ferlito et al., 2020).

Capacitance sensing bioelectronics is well-established. For example, it is used in the biopharmaceutical industry to improve scale-up cell culture processes (Konakovsky et al., 2015; Reinecke et al., 2017; Metze et al., 2020). This includes using capacitance-sensing probes to study and detect biomass and viable cell concentrations in suspensions and to determine how close to confluency a batch is. However, these use cases typically do not include adherent cell lines and much less the ability to detect cell counts from capacitance measurements.

Lab-on-CMOS capacitance sensors have also been demonstrated previously (Forouhi et al., 2019). They are advantageous because they allow smaller sample volumes to be analyzed. And, a plurality of analysis functions and signal processing can be integrated on the CMOS chip, which makes these microsystems ideal for point-of-care applications. Their use has been shown in drug cytotoxicity assays (Nabovati et al., 2019), potency assays for chemotherapeutic agents (Senevirathna et al., 2019a), viral infection assays (Abdelhamid et al., 2022), oral cell analysis (Osouli Tabrizi et al., 2022), and nanoparticle-mediated activation of neutrophils (Bunnfors et al., 2020).

Furthermore, the cell coverage of an electrode and its relationship with the elicited capacitance change at the electrode has been studied in order to quantify the relationship between measured capacitance and cell density in a capacitance sensing lab-on-CMOS device (Senevirathna et al., 2019a). For example, Senevirathna et al. showed that the measured capacitance at an electrode was correlated with the cell coverage at that electrode (B. P. Senevirathna et al., 2019b). Further, Renegar et al*.* developed a framework based on deep neural network image segmentation techniques to study the correlation between the measured capacitance and the coverage of single cells at an electrode (Renegar et al., 2022).

These works offer compelling evidence that biophysical phenomena (e.g., cell spreading and coverage) modulate interfacial capacitance. Furthermore, we have previously shown that the culture conditions that modulate cell proliferation rates yield differential capacitance responses. For example, we have shown cells cultured with growth rates inhibitors such as chemotherapeutics (Senevirathna et al., 2019b) and tumor-treating fields (Gilpin et al., 2022).

In the present study, we demonstrate a methodology for deriving a temporal model that can track the evolution of the number of cells in a wide area based on an average capacitance inferred from measurements originating from a plurality of electrodes disposed inside the area. This is unlike the aforementioned studies, which focused on local effects at the electrode sites. In contrast, we show herein that in sustained cell culture growth conditions, the set of sensing electrodes can be considered as one electrode registering an average capacitance growth factor over the course of the culture. We further show that this average capacitance growth factor and the cell counts registered over the area can be linked via a temporal model that tracks the evolution of the cell numbers inside the area of interest. This model and the data analysis techniques featured in this paper lay the groundwork for a generalized framework for gaining insights from capacitance sensors using multiple electrodes over a wide sensing area.

## 2 Materials and methods

### 2.1 Lab-on-CMOS biosensing platform

**FIGURE 1:**
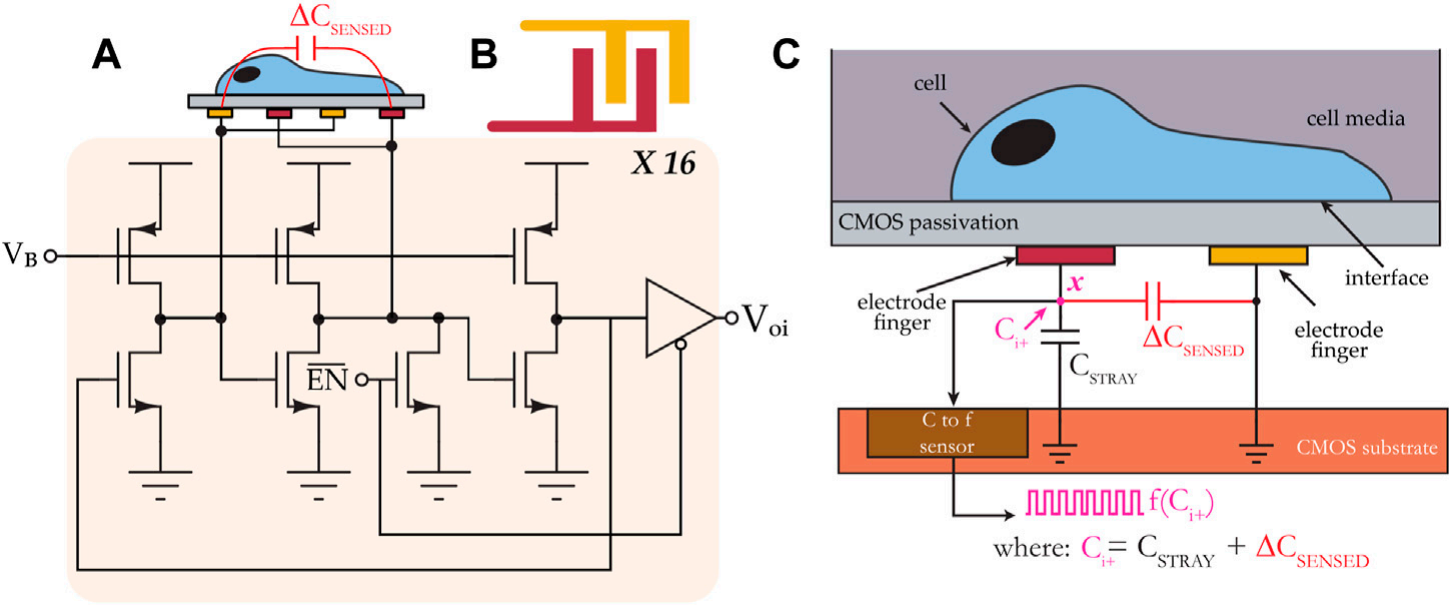
**(A)** System architecture of a CMOS capacitance sensor. The sensor consists of 16 individually-addressable capacitance-to-frequency pixels, and each pixel includes a ring oscillator circuit. **(B)** Top view of an interdigitated electrode. **(C)** Cross-sectional view showing the sensor’s transduction mechanism.

The biosensing platform used in this study included a microsystem configured for measuring cell proliferation and migration (Senevirathna et al., 2016; Senevirathna et al., 2019c; Gilpin et al., 2022). At its core, the microsystem included an application-specific integrated circuit (ASIC) fabricated in a 0.35 µm CMOS technology. The ASIC chip included 16 capacitance-to-frequency (CTF) sensors structured in a 4 × 4 array of integrated biosensor pixels with a spatial pitch of 196 × 186 μm. Each pixel included an interdigitated electrode structure with a sensing area of 30 × 30 μm^2^ and dedicated circuitry for transducing cell adsorption, movement, or life cycle events (e.g., mitosis) into an electrical signal. The electrodes were isolated from the cell media using the CMOS process’ native passivation layer. The sensors measure the capacitance at the cell interface, and they generate a digital signal that is outputted to an off-chip microcontroller via an on-chip I^2^C interface. The sensors’ principle of operation is described below, referring to Figures 1A–C.

The input capacitance to the electrode is labeled ∆C_SENSED_, and it consists of two capacitances. The first is the electrode’s intrinsic capacitance, which is variable, and the second is a fixed parasitic capacitance that exists between the electrode and a reference node of the circuit. The effective capacitance C_i+_ at node ***x***, which consists of the parallel combination of the two aforementioned capacitances, is mapped to a frequency f (C_i+_). In a cell assay using this sensor, for example, during the sedimentation phase when a cell gets close to the interdigitated electrode, ∆C_SENSED_ changes from its baseline value as a result of perturbations in the coupling field established by the electrode fingers; these perturbations change the electrode’s intrinsic capacitance. The frequency of the test signal is continually monitored, and it is used to calculate a relative change in capacitance, and this change in capacitance is subsequently correlated with cell activity.

### 2.2 System integration and experimental setup

The microsystem included an integrative package capable of maintaining the chip’s electrical integrity while allowing liquid samples to be applied onto its sensing surface. The packaging procedure employed was described in previous publications (Dandin et al., 2009; Datta-Chaudhuri et al., 2014; Gilpin et al., 2022). Briefly, it included attaching the ASIC die directly onto a PCB daughter board, which included a redistribution pad frame. Once attached to the PCB, the die’s I/O pads were wire-bonded directly to the leads of the redistribution pad frame. The wire-bonds were encapsulated with an impermeable epoxy. A subsequent encapsulation step was employed, extending the encapsulant from the edge of the chip to the edge of the redistribution platform in order to make a platform onto which additional structures could be built. A cell culture-compliant dish was then glued to the resulting epoxy platform. Within the cell culture dish, a second chamber was made using a 1 cm spectroscopy cuvette. Two holes were machined at the base of the cuvette in order to allow fluid exchange between the cuvette and the outer dish. The cells were plated inside the cuvette, and cell media was used to fill the dish with enough fluid to cause the cuvette to be filled to the brim, at which point a glass cover slip was placed on top of the cuvette.

This two-chamber arrangement was used in order to minimize fluid evaporation over the sensing area as well as to maintain a constant focus for imaging (Senevirathna et al., 2019a). The two-chamber arrangement utilizes no active medium perfusion. Rather, the setup is merely a culture well that includes an inner compartment and an outer compartment. The ports allow the medium to fill up both compartments. The inner compartment is filled to the brim, and it is subsequently covered using glass cover slip during the experiment. This approach provides the benefit of having a smaller evaporation rate in the inner compartment relative to the evaporation rate in the outer compartment. Because the region of interest is inside the inner compartment, this means that during the experiment, the optical path seen by the microscope does not change rapidly due to medium evaporation.

**FIGURE 2:**
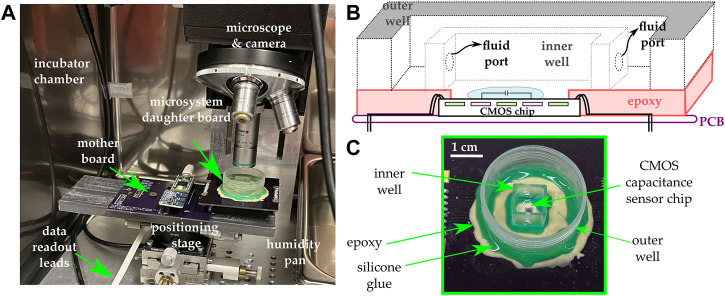
**(A)** The experimental setup includes an incubator maintained at 37°C and 5% CO_2_ and in which a microscope and a camera are mounted. The microsystem is mounted on a daughter board and subsequently connected to a mother board that includes a microcontroller and additional ancillary circuits for data acquisition and control. **(B)** Cross-sectional view of the integrative package used in the study. A two-well system is used in order to maintain a constant focus and minimize evaporation. **(C)** Photograph of the microsystem.

All experiments were conducted in a cell culture incubator at 37°C and under 5% CO_2_. A bright field upright optical microscope was placed inside the incubator to monitor the cells on top of the chip. Microscopy images were obtained every 5 min over the duration of the experiment (typically 24–48 h) using a C-mount digital camera attached to the microscope. The data acquired from the chip and the images were automatically uploaded to a decentralized cloud environment where image processing and data analytics were performed using custom algorithms. Figure 2 illustrates the various components of the system.

### 2.3 Cell culture

Murine Balb/c Raw 264.7 macrophages from an immortalized cell line were purchased (ATCC) and cultured in a T75 flask with culture media containing DMEM (ThermoFisher Scientific) without phenol red and with a pH buffer (HEPES) having 10% FBS (VWR). Culture media was removed from the T75 flask, and 7 mL of sterile PBS was added to the culture flask and incubated for 5 min to wash the cells. The PBS was removed, and 7 mL of media was added to the flask. A cell scraper (VWR) was used to resuspend the cells in the media. A volume of 5 mL containing the cells was added to a 15 mL centrifuge tube and centrifuged for 5 min. The supernatant was removed, 2 mL of fresh media was added to the centrifuge tube, and the cells were resuspended and added to a new T75 flask with 8 mL of fresh media. From a mixture having an estimated cell density of 2,80,000 cells/mL, we pipetted 7 mL of the cell solution so that the starting number of cells seeded in the microsystem was ∼2 million. We note that upon seeding the cells at that density in the microsystem’s analysis well, only a small fraction of the starting cell population fell in the area spanned by the electrode array, and the starting number of cells around the electrode array was sufficient to register sustained cell culture growth. As such, for each of the five experiments featured in this paper, the starting cell density of 280,000 cells/mL was used to ensure that there were enough cells in the sensing area to conduct our proliferation experiments over a 30-h period.

### 2.4 Data collection and analysis

The data yielded by the experiments consisted of a time series dataset originating from measurements from the 16 sensors comprised on the chip and of an imaging dataset originating from micrographs acquired by the camera attached to the microscope. The time series data consisted of capacitance change measurements (ΔC) from all the electrodes, performed every 29 s. As noted previously, the imaging dataset was generated by taking an image of the cell culture every 5 min. Both datasets were time-stamped automatically by the control program, and experiments were typically conducted for 48 h. Exemplary data from each dataset are shown and discussed in detail in the following subsections and in the Supplementary Information document accompanying the paper.

#### 2.4.1 Imaging dataset

**FIGURE 3:**
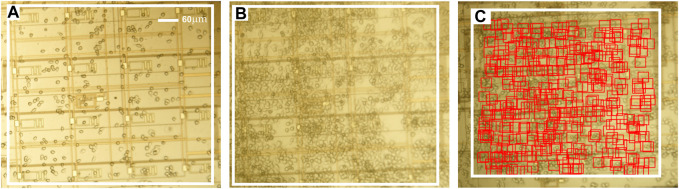
**(A,B)** Photomicrographs of the microchip at two different times after macrophages are plated thereon in a cell medium. **(C)**. Markers showing cells identified by the computer vision code used to estimate the number of cells in the ROI. Additional details about the computer vision code are provided in the Supplementary Information document accompanying the paper.

Figure 3 shows a subset of post-processed images from the imaging dataset, with a white box enclosing the region of interest (ROI), i.e., the sensing area spanned by the 4 × 4 electrode array. Prior to cell counting, the images were post-processed using image processing packages available in python (Open CV, matplotlib, and numpy). Post-processing included contrast enhancement, sharpening, and applying a false color scheme. These post-processing steps were conducted to enhance image quality in order to better visualize the cells adhered to microchip’s surface and further to facilitate the automatic detection and counting of cells using a custom-designed Python algorithm for cell identification and pattern classification. This algorithm is further described in detail below.

**FIGURE 4:**
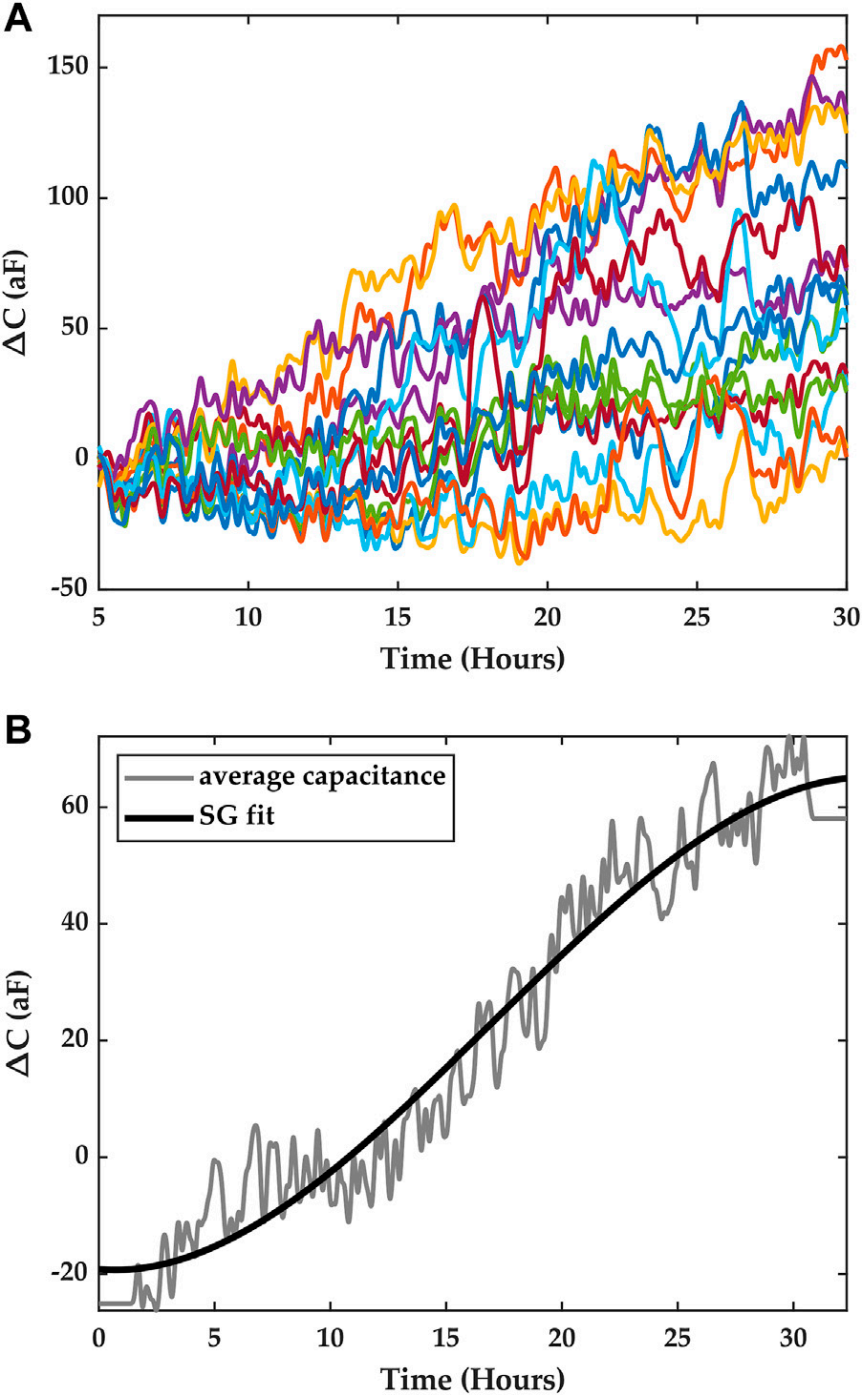
**(A)** Time series data obtained from a macrophage growth experiment. The traces corresponds to the measurements from the sensor array, and each color corresponds to one electrode in the ROI. **(B)** Average capacitance data (gray) were obtained by averaging the measurements from all the pixels. The solid black trace is a trend-preserving fit that is obtained using a Savitsky-Golay filter.

Cell counting was effected using a custom template matching algorithm developed in Python. Briefly, the algorithm first included post-processing the images of the dataset, as described above. Second, using a reference image with cells present in the ROI, a template was extracted by overlapping each cell in the image with its center aligned to the others and taking an average intensity. The template served as a correlation filter which the algorithm attempts to retrieve in subsequent images in the dataset. When a high correlation between a feature in an image and a template was obtained, the algorithm flagged the location as likely having a cell, and it marked the image with a graphical indicator (e.g., a red square, as shown in Figure 4) at that location (Kim and de Araújo, 2007; Bolme et al., 2009). To count the cells in the image, the algorithm reported the number of indicators registered upon parsing the ROI on a pixel-by-pixel basis. A result of the cell counting algorithm is shown in Figure 3C for one representative image.

#### 2.4.2 Time series dataset

The time series data set included measurements from the capacitance sensors comprised in the array. A measurement was taken every 29 s, yielding 16 data points (one for each electrode), and for the duration of the experiment. The change in capacitance was calculated by subtracting an initial measurement of capacitance prior to the start of the experiment from each of the instantaneous measurements obtained during the experiment. Figure 4A.) shows the resulting measurement traces across the array for the macrophage growth experiment depicted in Figure 3.

Contrary to previous works on integrated capacitance data for cell measurements where cell coverage of the electrode was correlated with the measured capacitance, in this study, we consider the average capacitance obtained from all the electrodes in the array and seek to correlate it with cell *growth*. We argue that this average capacitance can be considered as an overall indicator of cell growth in the ROI, where each electrode serves to sample the ROI spatially, and we seek to establish a temporal model that links cell numbers and the average capacitance.

Furthermore, to obtain a capacitance curve that characterizes capacitance growth in the ROI for a given period of time, we utilize the trend of the data rather than instantaneous average capacitance data. This means local temporal variations in capacitance due to Brownian motion, cell movements in the vicinity of the electrodes, and detector noise are averaged out. To obtain this trend, we fitted the average data with a Savitsky-Golay (SG) filter of the third order, with a frame length equal to *n-1*, where *n* is even and is the number of points in the average capacitance time series (Savitzky and Golay, 1964). Figure 4B shows the results of the fit, which were obtained using Matlab. This fit captures the trend of the capacitance registered in the ROI over the course of an experiment for a period of ∼30 h.

To compute a single capacitance growth metric, one may simply perform a piece-wise linear approximation of the SG fit and estimate therefrom a capacitance growth factor *S* (in ΔC/hour) for each segment, *S* being the slope of the segment. An average capacitance growth parameter *S*_*avg*_ may be computed by averaging all the slopes. The piece-wise linear approximation may be performed by dividing the SG trace in 1-h overlapping segments, with a half-hour overlap between segments. Subsequently, a linear regression may be conducted on each segment to estimate the average capacitance growth factors *S,* from which Savg may be computed. Alternatively, a histogram of all the slopes calculated from SG fits of each pixel trace may be constructed, and the mean value of the resulting distribution can be used as the average capacitance growth parameter *S*_*avg*_ *.* The standard deviation of the distribution provides an appreciation for the deviation away from the mean capacitance growth factor registered during the experiment.

**FIGURE 5:**
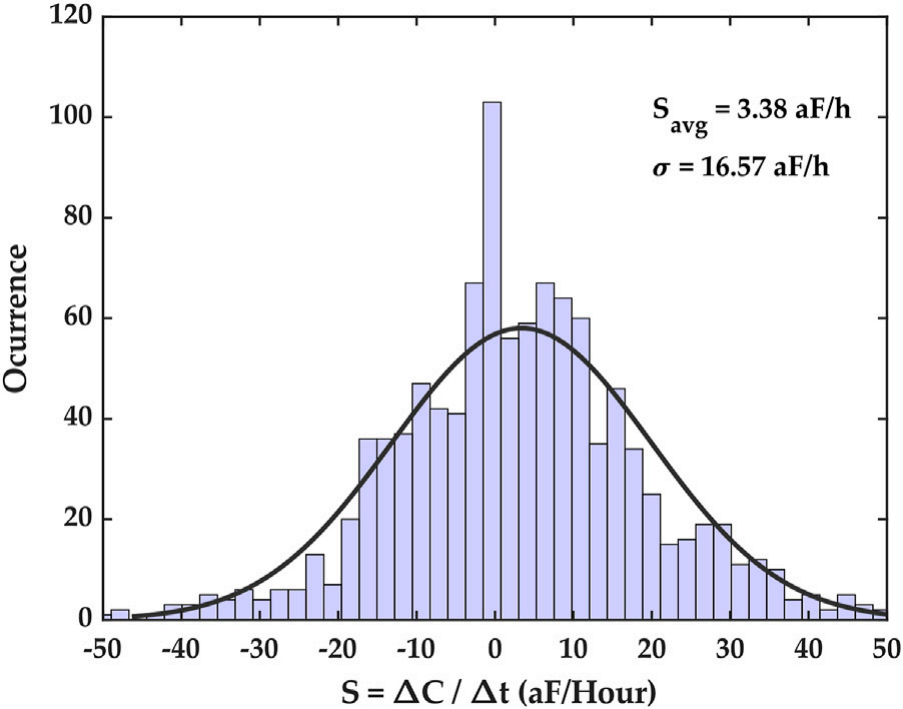
Capacitance growth factor (S) histogram for the time series capacitance dataset. This histogram shows the extent of capacitance growth factors over the entire electrode array as well as the number of occurrences of measured capacitance growth factors in the dataset. The distribution’s mean is *S*_*avg*_ = 3.38 aF/h, with a standard deviation of *σ* = 16.57 aF/h. The parameter *S*_*avg*_ is the average capacitance growth registered during the experiment.

The latter method was used, and its results are shown in Figure 5. The capacitance growth factors obtained for the entire dataset were binned to form a histogram that highlighted the diversity of capacitance growth factors across the ROI, and the mean of this distribution provided an average capacitance growth factor estimate over the ROI, denoted *S*_*avg*_. For the featured dataset, the average capacitance growth factor *S*_*avg*_ was 3.38 aF/hr, with a standard deviation of 16.57 aF/hr. Here we assume that the distribution may be approximated as a normal distribution with the same mean and standard deviation. Our rationale for this assumption, along with normality tests are reported in the Supplementary Information document accompanying this paper.

## 3 Results

**FIGURE 6:**
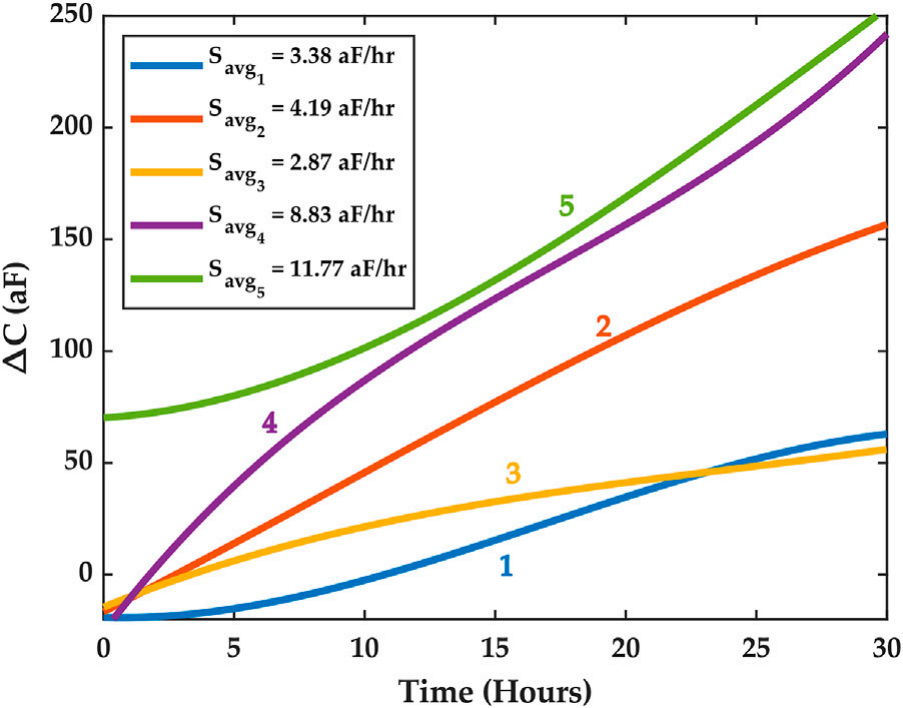
SG trends for five experiments. The high cell culture growth phase is assumed to be within the first 30 h. Different average capacitance growth factors *S*_*avg*_ were registered across the five experiments. The experiments are labeled 1 through 5 and color is used to distinguish them. The Supplementary Information document features an experiment where cell medium only was used, and this experiment revealed no increase in average capacitance as shown here for the five macrophage proliferation experiments.

We conducted several growth experiments and analyzed their results using the methods discussed above. We feature herein five representative experiments where macrophages were cultured on the chip and left to proliferate for 48 h. The first 30 h of each experiment were considered for studying macrophage growth dynamics and for correlating measured capacitance results with cell counts estimated via imaging. This time frame was chosen empirically based on observations that the ROI would saturate with cells beyond 30 h, thus resulting in a reduced cell growth rate in the ROI. Figure 6 illustrates the average SG fits for each of the five experiments starting from t = 0 to t = 30 h.

**FIGURE 7:**
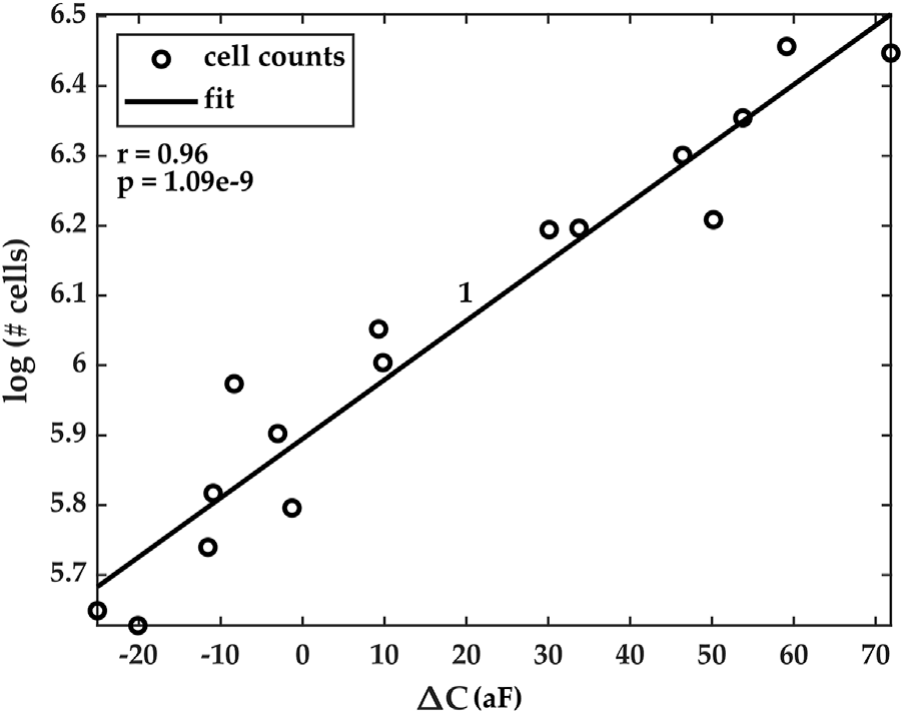
Correlation plots of estimated cell counts (in natural logarithm scale) and the measured average capacitance. Pearson’s correlation coefficients *r* and its corresponding *p*-value for Experiment 1 are r = 0.96 and *p* < 10^–8^. Correlation plots for Experiments 2–5 are reported in the Supplementary Information document accompanying the paper.

Cell counts were estimated for each experiment using our custom Python code. To do so, a subset of images from the imaging dataset was chosen. The time stamps for each image were extracted, and the number of cells in the ROI was estimated by the algorithms. Furthermore, the values of the average capacitance data at the same time stamps were extracted from the time series dataset. Our results show that the natural logarithm of the cell counts was highly correlated with the average capacitance and that the correlation was linear. Figure 7 shows the correlation results for Experiment 1. Similar results were obtained for Experiments 2–5, but they are omitted here for conciseness. The data for these additional experiments are reported in the Supplementary Information document accompanying this paper.

This finding, specifically, that the logarithm of cell counts and average capacitance follow a linear relationship, lays the foundation for a time-dependent model that can be used to predict future cell numbers or to calculate the instantaneous number of cells in the ROI, after sufficient capacitance measurements and images have been collected.

To construct a temporal model that relates cell numbers and capacitance, we first used a linear regression to fit a line between the logarithm of the measured number of cells and the measured average capacitance data for the ROI, and we write Eq. 1, which captures this log-linear relationship. Here, *N*_*m*_ is the number of cells, *α* is the slope of the line, *ΔC*_*m*_ is the average capacitance of the ROI, and *K* is the intercept of the line.

$$\ln\left(N_{m}\right)=\alpha ∆C_{m}+K$$ (1)

$$\frac{\ln\left(N_{m}\right)}{t}=\frac{\alpha ∆C_{m}}{t}+\frac{K}{t}$$ (2)

$$S_{avg}=\frac{∆C_{m}}{t}$$ (3)

$$\ln\left(N_{m}\right)=\alpha S_{avg}t+K$$ (4)

$$e^{\ln\left(N_{m}\right)}=e^{\alpha S_{avg}t+K}$$ (5)

$$N_{m}={Ae}^{\alpha S_{avg}t},where\;A=e^{K}$$ (6)

Eq. 1 is normalized by the variable *t*, yielding Eq. 2, which introduces the time dependence in the model. Here, the implicit assumption that is made is that the log-linear relationship between the number of cells and the measured average capacitance is itself time-invariant. Specifically, we assume that neither *α* nor *K* has a time dependence. We will discuss in the following section situations under which this assumption fails and in which, consequently, the proposed model cannot accurately capture the temporal evolution of the cell population within the ROI.

In Eq. 3, we assume that the term *ΔC*_*m*_ */t* is the average capacitance growth factor *S*_*avg*_. As previously noted, the *S*_*avg*_ factor is a measure of the average rate of change of the capacitance for a normally distributed set of average rates across the ROI. Thus, our assumption here is justified since that for any given capacitance growth rate *ΔC*_*m*_ */t*, there is a roughly 70% probability that this rate is within one standard deviation of the mean of the distribution. This, of course, is because the distribution is assumed to be Gaussian, as shown in Figure 5. Lastly, there is another caveat. For distributions with large standard deviations, Eq. 2 is less likely to hold. However, we posit that datasets with large standard deviations would fall in the category for which the model would not hold anyway, as we shall discuss later.

Equations 4, 5 show the intermediate steps that lead to Eq. 6, which broadly states that the number of cells in the ROI follows an exponential dependence time, and where the argument of the exponential includes experimentally determined constants *α*, *A*, and *S*_*avg*_. Predictably, simply fitting Eq. 6 to the measured cell counts as a function of time does not work. This is readily observable when one considers that the constant *A* depends on the *y*-intercept of the linear fit. As such, at t = 0, Eq. 6 is likely to predict the cell count erroneously. In other words, the intercept may differ enough from the actual value and bias the exponential away from the measured data.

Thus, we posit that Eq. 6 is a generalized temporal model that links measured capacitance growth factor and measured cell numbers. Consequently, Eq. 6 must be updated further in order to obtain an experiment-specific model that depends on independent variables that are particular to the experiment. These independent variables consist of time, which is already captured in Eq. 6, and two other independent variables which are the cell numbers at two specific times. The latter two variables form boundary conditions for Eq. 6. The first boundary condition is *N*_*0*_, *i.e.,* the number of cells at time *t*_*0*_, and the second boundary condition is *N*_*f*_, the number of cells at time *t*_*n*_, where *n* is strictly positive. The particular model is formulated in Eq. 7. The constants *Γ* and *β* can be determined using the boundary conditions shown in Eqs 8, 9, provided *N*_*0*_ and *N*_*f*_ are known. The parameters *α* and *S*_*avg*_ are determined experimentally using the imaging and time series datasets for the time interval (*t*_*0*_ *, t*_*n*_).

$$N_{m}={\Gamma e}^{\alpha S_{avg}t}+\beta$$ (7)

$$\Gamma e^{\alpha S_{avg}t_{0}}+\beta =N_{0}$$ (8)

$${\Gamma e}^{\alpha S_{avg}t_{n}}+\beta =N_{f}$$ (9)

**FIGURE 8:**
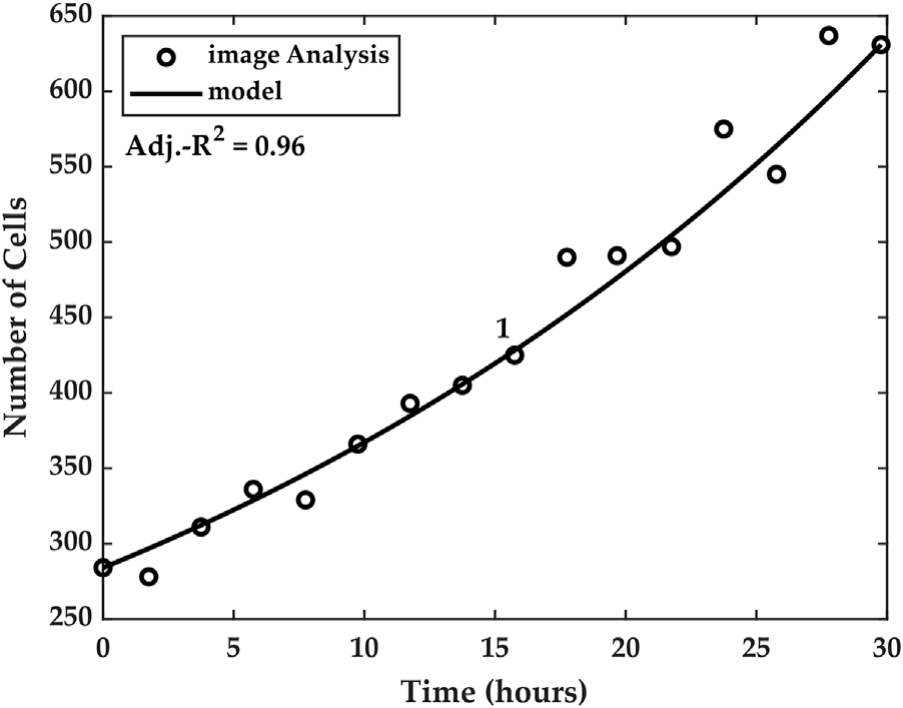
Temporal model linking cell numbers and capacitance. The model utilizes the average measured capacitance growth *S*_*avg*_ to account for the cell numbers at time *t* ∈ (*t*_*0*_ = 0, *t*_*f*_ = 30) for Experiment 1. *t* ∈ Temporal model plots for Experiments 2–5 are reported in the Supplementary Information document accompanying the paper.

We tested the particular temporal model against the cell count measurements for each of the five experiments. Good agreement was found between the estimated cell counts and the temporal model, as can be seen in Figure 8, which shows the model for Experiment 1. The goodness of the model was evaluated using an adjusted-R^2^ coefficient of determination, where the number of independent variables was *k* = 3.

Table 1 summarizes the measured results for the five experiments. We report the *S*_*avg*_ parameters computed for each of the five experiments and the Pearson correlation coefficient and its associated *p*-value, which assess the correlation between measured capacitance and measured cell numbers. Further, we report the R^2^ and the adjusted R^2^ values of the proposed model. Table 1 also reports the doubling time calculated from the model.

**TABLE 1:** Summary of experimental results and statistics for the five experiments. The last column of the table shows the estimated doubling time *t*_d_ based on the proposed model. In Experiment 5, the cells did not double in number inside the ROI during the 30-h period.

*Experiment ID*	*S*_*avg*_(aF/hr)	*σ* *(aF/hr)*	*r*_*c, n*_	*p-value*	*R* ^*2*^	*adj. R* ^*2*^	*t*_*d*_ *(hr)*
***1***	3.38	16.57	0.96	1.09E-9	0.97	0.96	26
***2***	4.19	20.43	0.96	5.11E-10	0.83	0.79	20
***3***	2.87	25.15	0.94	1.66E-6	0.93	0.91	21
***4***	8.83	10.53	0.94	8.31E-7	0.93	0.91	28
***5***	11.77	7.49	0.96	1.61E-8	0.87	0.83	—

We caution here that our measured doubling time may not equate to the cell line’s doubling time. Specifically, our measured doubling time only accounts for the time it takes for the initial number of cells *inside the ROI* to double, and it is calculated based on our temporal model and confirmed by the imaging data. Therefore, it only accounts for the measured activity inside the ROI, and thus it is not a measure of the cell line’s characteristics. In other words, because the number of cells in the ROI is small (e.g., there are only a few hundreds to a thousand cells in the ROI, from the two million that were seeded initially), the doubling time estimated with such a small sample lacks the statistical significance to conclude that the cell line would have the same doubling. Rather, our measurement merely provides a metric for analyzing the cell number evolution inside the ROI during the experiment. This number varies from experiment to experiment, and, for example, as shown in Table 1, in some cases, the number of cells in the ROI does not double during the observation period (Experiment 5).

## 4 Discussion and conclusion

This study demonstrated a system configured for tracking macrophage proliferation in real time in an area spanned by multiple capacitance sensing electrodes *in situ* during their culture on an electronic chip. While cell culture on electronic chips has been shown before, particularly on capacitance sensing chips, several novel contributions stem from our work. We demonstrate that a set of electrodes that are sparsely disposed within a region of interest can serve to monitor cell proliferation kinetics temporally over the entire region. Specifically, we show that an average capacitance growth factor measured by all the electrodes in the area is a linkage factor for a model that relates cell numbers to measured capacitance. This model (shown in Eqs 7–9 accurately tracks cell proliferation in the ROI, provided that the number of cells is known at two distinct times.

One of the model’s key determinants is the degree of correlation that exists between the measured cell counts and the average capacitance when estimating the parameter *α* in Eq. 7 *.* Recall that this parameter is the rate of change of the number of cells as a function measured capacitance, and we assumed that it was constant and time-invariant, and thus that capacitance and the logarithm of the cell numbers were linearly correlated. These conditions must be met in order to ensure that the temporal model accurately describes cell culture growth in the ROI.

However, there are several conditions under which the degree of correlation between average capacitance and cell numbers may be reduced, which would reduce the accuracy of the model shown in Eqs 7–9. For instance, when the ROI experiences non-negligible cell migration, whether in or out of the ROI, there may be a non-linear correlation between measured capacitance and measured cell numbers. This scenario would likely invalidate the assumption that the parameter *α* is constant and time-invariant, and Eqs 7–9 would thus not be adequate to describe the cell dynamics within the ROI. Such a scenario may cause a larger spread around a mean capacitance growth factor, since electrodes that are closer to the migration sites in the ROI may experience a much greater or much lower average growth factor, depending on the direction of the migration.

In yet other situations, the correlation between the two measured variables may be reduced. For example, the degree of sparsity of the electrodes inside the ROI may significantly reduce the correlation between the observed capacitance and the number of cells inside the ROI. This is because the farther apart the electrodes are, the more localized their individual responses are. As such, measured capacitance would only correlate with small groups of cells that are in the vicinity of the electrodes. Conversely, in ROIs that are densely populated with electrodes, it is more likely to obtain high correlations, and thus, this should be a design goal in future capacitance-sensing lab-on-CMOS devices. However, increasing electrode density has practical implications for performance: the closer electrodes are placed, the more a single electrode serves as a parasitic load to its nearest neighbors. This would reduce electrode sensitivity. As such, there is a density-to-sensitivity trade-off that must be mitigated at the circuit design phase.

The lab-on-CMOS platform featured in this paper is suited for label-free studies of two-dimensional cell cultures. It has been used in two-dimensional cell studies for quantitatively characterizing the potency of external stimuli on cells under study. For instance, *Senevirthna et al.* showed a capacitance-based study for assessing the potency of a chemotherapeutic agent (cisplatin) on two types of ovarian cancer cell cultures, with one culture featuring cells that were sensitive to the chemotherapy and the other cells that were less sensitive to it. The study revealed a differential capacitance response between the two cultures, indicating a difference in proliferation activity, consistent with the respective sensitivities of the two types of cells to the chemotherapy. Furthermore, Gilpin et al*.* showed that capacitance sensing could be used to monitor the real time effect of tumor-treating fields (TTFields) on breast cancer cells, thereby providing a simple tool for conducting efficacy studies of different TTField regimens on breast cancer cells. And, *Bunnfors et al.* showed that capacitance sensing could be used to track the nanoparticle-triggered activation of human neutrophil granulocytes.

The present work will augment the capabilities of capacitance sensor systems by providing a sensor analytics framework that can be integrated in hardware or software as part of a lab-on-CMOS digital signal processing (DSP) core. In this context, the proposed model may be used periodically and selectively, i.e., on chosen time segments during data acquisition, to predict short-term cell populations swings.

## Data Availability

The raw data supporting the conclusion of this article will be made available by the authors, without undue reservation.
