# Supplementary material for: Measuring and modeling macrophage proliferation in a lab-on-CMOS capacitance sensing microsystem
Source: Front Bioeng Biotechnol. 2023 May 12;11:1159004. doi: 10.3389/fbioe.2023.1159004 (PMC10213696; doi:10.3389/fbioe.2023.1159004)
Supplement: Supplementary file 1 [file DataSheet1.PDF]

Measuring and Modeling Macrophage Proliferation in a Lab-on-CMOS Capacitance Sensing Microsystem

Supplementary Information

Kyle Smith[1], Ching-Yi Lin[2], Yann Gilpin[2], Elizabeth Wayne[1,3]*, and Marc Dandin[2,3]*

[1]Department of Chemical Engineering,
[2]Department of Electrical and Computer Engineering,
[3]Department of Biomedical Engineering,
Carnegie Mellon University, Pittsburgh, Pennsylvania, USA

1 Introduction

In this document, we provide experimental details pertaining to the study featured in the main paper. We cover the hardware and software tools that were used. The software is in a publicly accessible repository on IEEE Dataport[1] and in our Google Drive[2]. Our discussion also features results from four representative experiments, in addition to the one experiment featured extensively in the main paper.

2 Detailed Description of the Experimental Methods Used in the Study

2.1 Hardware

Our hardware testbed included a cell culture incubator (Figure 1) whose chamber conditions were set and maintained at 37° C and 5% CO_2 for the duration of our experiments. The incubator was a Thermo Scientific Forma Series 3 184L water-jacketed CO_2 incubator. Further, the incubator featured an in-chamber microscope – a MicroZoom II bright-field up-right microscope from Bausch and Lomb. The objective used was an 8X objective, and the approximate working distance (*i.e.*, the distance between the top of the objective and the chip's surface) was 2 cm. The microscope was equipped with an 18 mega-pixel C-mount digital camera (MU1803, Amscope, United Scope, CA). Data from the camera were routed to a PC located outside of the incubator. To do so, we used a flat USB cable (UOGW, 20FT USB-A to micro-USB) such that the cable could go outside the incubator door without compromising its seal.

The chip was mounted on a daughter printed circuit board (PCB), and the chip's input/output pads were connected directly to the daughter PCB's signal traces via wire bonding. The bond wires were encapsulated using a thermally conductive epoxy (Cotronics, Durapot 865). Chip mounting, wire bonding, and encapsulation were performed by Micro-Precision Technologies Inc., Salem New Hampshire. Following a second encapsulation step with the same epoxy and conducted in-house (Gilpin *et al.*, 2022), a culture dish was created from cutting a 3.8 cm diameter centrifuge tube and gluing it on the board. This culture dish was 1.6-cm tall, and it formed an outer well in a two-well culture system (Senevirathna *et al.*, 2019). The inner well was made by cutting a 1 cm optical

---

[1] https://dx.doi.org/10.21227/h4bm-ac75

[2] https://drive.google.com/file/d/1ncHIz2yKT23G1kMgKJY1VEvQASzNWxjb/view?usp=share_link

spectroscopy cuvette and drilling two holes at the bottom approximately 5 mm from its base. The holes were nominally 2 mm in diameter. This cuvette was glued at the center of the outer culture well, and it was made to surround the chip equally on all sides. The glue used was a medical-grade biocompatible silicone (Kwik-CAST, World Precision Instruments, Sarasota, FL). During experimentation, the cuvette forming the inner well was covered with a glass cover 22 mm x 22 mm cover slip (1404-15 Globe Scientific Mahwah, NJ).

The culture apparatus described above is merely a culture well that includes an inner compartment and outer compartment. It uses no active perfusion of cell media. Rather, the holes, which form communicating ports between the two compartments, allow the medium to fill up both compartments, and the experiments are conducted in their entirety with the starting volume of cell media, *i.e.*, without media replenishment. During our experiments, the inner compartment was filled to the brim, and it was subsequently covered using the glass cover slip. This approach provides the benefit of having less medium evaporation in the inner compartment relative to the evaporation in the outer compartment. In other words, while evaporation does occur in this two-well system, it does not occur at a rate which rapidly degrades the focus for the imaging when considering the inner compartment.

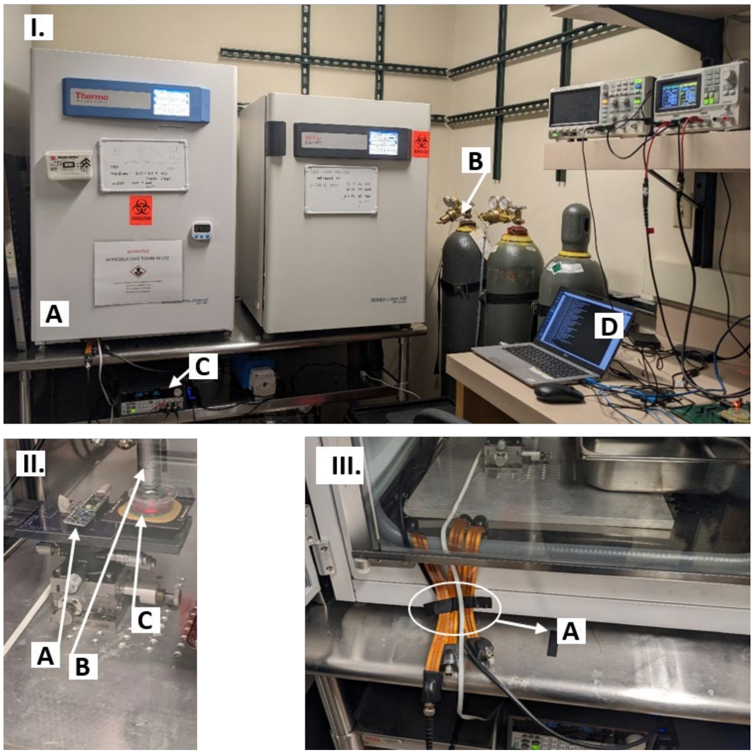

Figure 1. Experimental setup. (I.) The setup used a cell culture incubator (A) whose chamber was maintained at 37 ºC and at 5% CO_2. The CO_2 was provided via a tank (B) attached to the incubator using a dedicated line. The shelf under the incubator houses instruments for providing power to the microcontroller and the chip (C). Data from the chip is outputted via the incubator door to a PC (D). (II.) View of the incubator's chamber comprising our test device. Here (A) is a microcontroller used for chip control and readout, (B) the objective of the in-incubator microscope, and (C) the device itself, housing cells in a cell culture medium. (III.) This photo shows the flat cables (A) that were used, with the inner glass door of the incubator being closed.

Furthermore, because the region of interest is inside the inner compartment, the optical path seen by the microscope does not change significantly due to medium evaporation (Senevirathna *et al.*, 2019),

though, as we shall see below, some image blur is still incurred at later times in the experiment due to the minimal evaporation experienced.

An x-y stage (9067-XY-M, Newport Inc., Irvine CA) and tip-tilt and rotation stage (TTR001/M ThorLabs Newton NJ) were combined to create a 5-axis positioning stage that was placed under the microscope, and a mother PCB was affixed to the stage's platform using an optical screw. The mother PCB included ancillary circuits for operating the chip. Example ancillary circuits included a micro-controller (Teensy 3.2, PJRC, Sherwood OR), which generated control signals for the chip. The daughter PCB was interfaced to the mother PCB via sockets placed on the mother PCB. The mother PCB was connected to outside instruments using a set of BNC cables (CMP-FLT_BNC-GHST, The Cimple Co.). Again, to ensure that connections could be made to a PC and other instruments (e.g., power supplies) outside of the incubator, flat BNC cables were selected for our application.

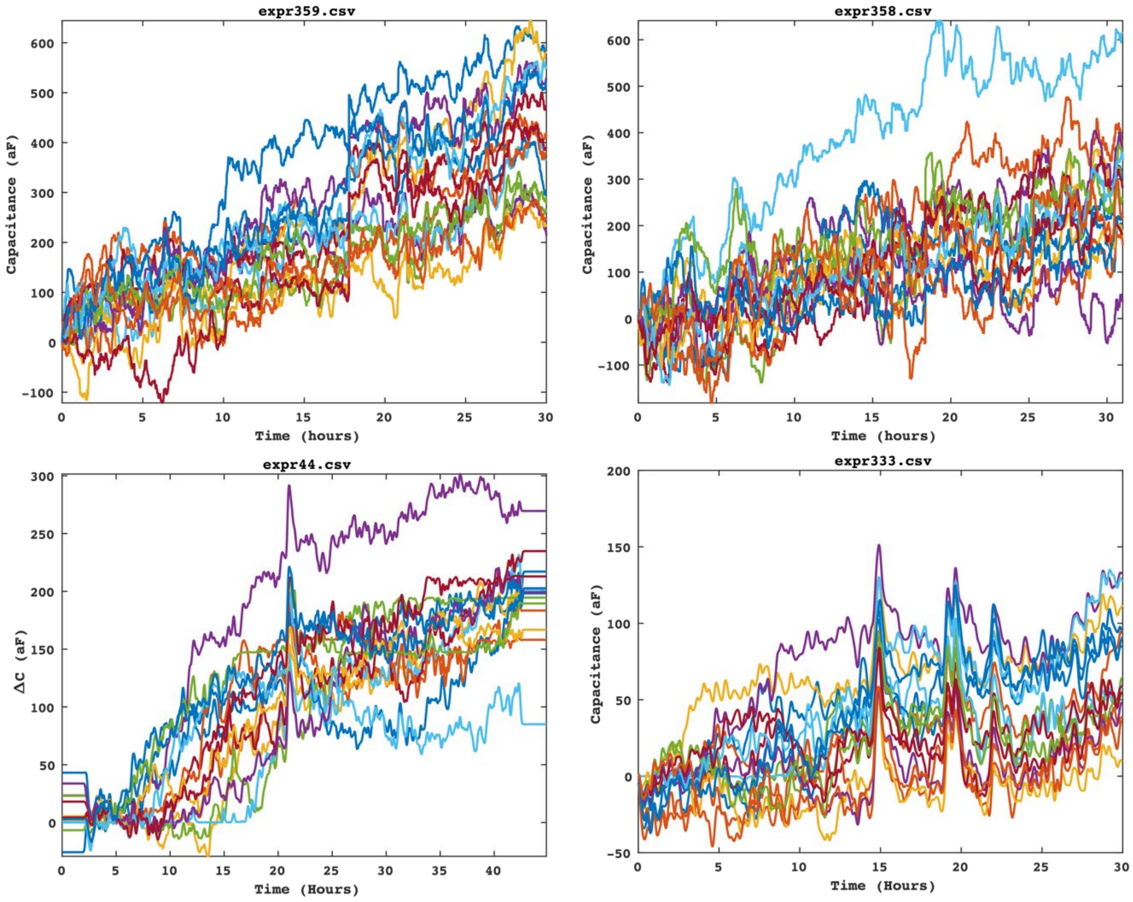

Figure 2. Traces extracted from the .csv measurement file and plotted for a 30-hour time window by the meanCap.m m-file. Each trace corresponds to one electrode in the ROI. Color is used to indicate different electrodes. The file names containing the data for each experiment is shown at the top of the corresponding charts. These files may be found in our online repositories.

2.2 Software

The software used in this study along with sample data files[3] with which the software may be evaluated are archived on IEEE Dataport and our Google Drive, as noted above. We described herein the software for the time series data processing featured in the paper, as well as the computer vision algorithm that was used for cell count estimation. The time-series software requires at least the R2019b release of Matlab (Mathworks, Inc., Natick, MA), and the computer vision algorithm requires Python 3.10.6 or newer.

2.2.1 Time Series Processing

The capacitance measures at each time instant are saved in a **.csv** file. The m-file loads the data, plots them, and computes a Savitksy-Golay fit using the **sgolayfilt** built-in Matlab function. Furthermore, **meanCap.m** uses **slopeCompute.m,** which computes the distribution of slopes measured across the ROI. Figure 2 shows the measurement traces plotted by **meanCap.m** for four experiments. The first five hours of each experiment are discarded to remove the capacitance transient that occurs after starting the experiment. Furthermore, the data between t = 21 and t = 24 are also discarded in order to remove a capacitance transient that occurs when the incubator door is opened to manually adjust the focus after image blur arises from the minimal evaporation experienced. After discarding these data, the x-axis is then remapped to show a continuous 30-hour observation time.

Figure 3 below shows the mean capacitance estimated using **meanCap.m** and the Savitsky-Golay (SG) fit obtained using the built-in **sgolayfilt** function for four experiments. The fit uses a third order filter over the mean capacitance data. The **meanCap.m** m-file calls the **slopeCompute.m** m-file, which is a function that constructs a histogram of average capacitance growth factors. The average capacitance growth factor is $\Delta C/\Delta T$, and it has units of aF/hour. It estimates the rate of change of capacitance for a given period. As we have shown in previous work published (Gilpin *et al.*, 2022), partitioning the dataset and generating a histogram of average capacitance growth factors across the 16 electrodes for a given time segment shows the distribution of measurements across the ROI during that time segment. This method allows the comparison of capacitance measurements from two different time segments; this is done simply by tracking shifts between the means of the two distributions.

In the present study, we extend this approach, and we construct a histogram from a set of segments obtained by partitioning the thirty-hour time window in equal portions. The **slopeCompute.m** m-file computes the SG fit for each trace (i.e., for each time series from the 16 electrodes), and it subsequently estimates the slopes of a set of non-overlapping, equal, and consecutive segments over the 30-hour time frame. This is done for each of the 16 SG fits. All the slopes are then binned to create the histograms shown in Figure 4; the segment length was ~ 5 hours, yielding 6 consecutive segments. The resulting histogram is a mixture distribution of the individual segment slope distributions across the 16 traces.

Here, we show that the sub-populations (*i.e.*, the single-segment slope distributions) that make up the histogram of Figure 4 are normally distributed. To test for normality, we used the Shapiro-Wilk (SW) test. In the SW test, the null hypothesis is that the data under test are sampled from a normal distribution of unspecified mean and variance. A p-value of greater than a significance level α means that we cannot discard the null hypothesis. In other words, for a p-value greater than α, the data are likely sampled from a normal distribution. In our test, we used $\alpha = 0.05$.

[3] See Section 2.5 for an exhaustive list of the files and resources that are provided with the Supplementary Information.

Table 1. Normality tests for the distribution of average capacitance growth factors over various time segments and for the distribution shown in Figure 4.

Shapiro-Wilk Tests (α = 0.05) (expr359.csv)								
	S1	S2	S3	S4	S5	S6	30-hr	histogram
p-value	0.1528	0.7021	0.3727	0.9718	0.0539	0.1882	0.3105	0.0012

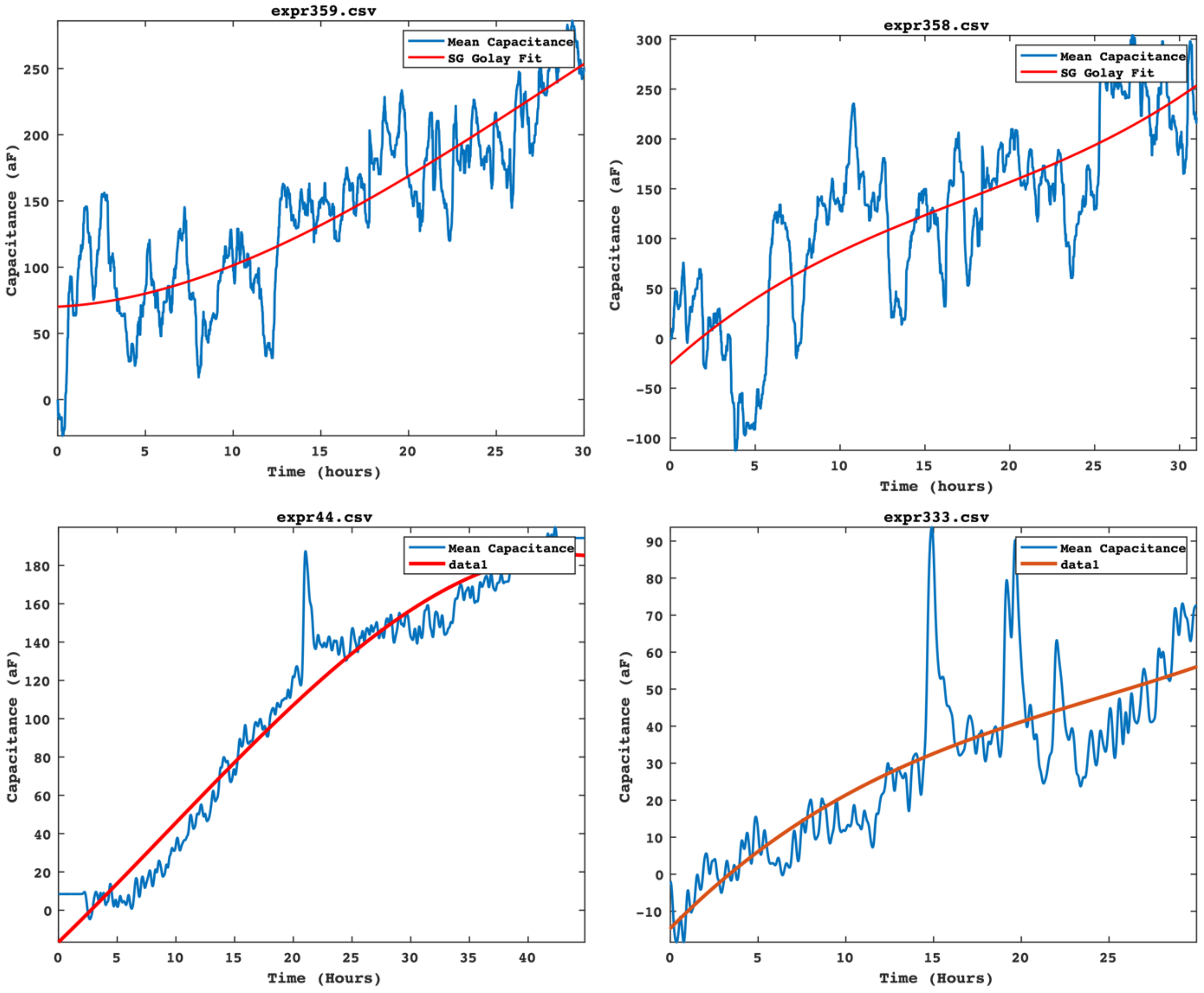

Figure 3. Mean capacitance data reported by meanCap.m for four experiments. The corresponding Savitsky-Golay fits are shown for each experiment.

Table 1 shows the SW test results for the 6 segments (S1-S6) and for a single segment of 30 hours. Furthermore, it shows the results for the mixture distribution. Specifically, the p-value for the histogram is under the significance level, which means that the mixture distribution is not normal.

However, in every other case, the p-value exceeds the significance level, which means that we cannot discard the null hypothesis and that these data are sampled from a Gaussian distribution. Furthermore, in the limiting case, *i.e.*, when we choose the segment length to be 30 hours, the distribution is also found to be normal. This implies that measures of average capacitance growth are normally distributed over the ROI during the experiment.

The mean of the 30-hour distribution was 12.7 aF/hour and the standard deviation was 4.2 aF/hour. The mean and standard deviation of the mixture distribution were 11.3 aF/hour and 8.3 aF/hour, respectively. Because the two means are close, we assumed that the mean of the mixture distribution can be used as a measure of the average capacitance growth over the 30-hour period. We denote this approximate measure as S_{avg} in the temporal model featured in the paper. Generally, we assume that the mixture distribution can be approximated with a Gaussian distribution having mean S_{avg} and standard deviation σ_{avg}, the latter parameter being the standard deviation of the mixture distribution.

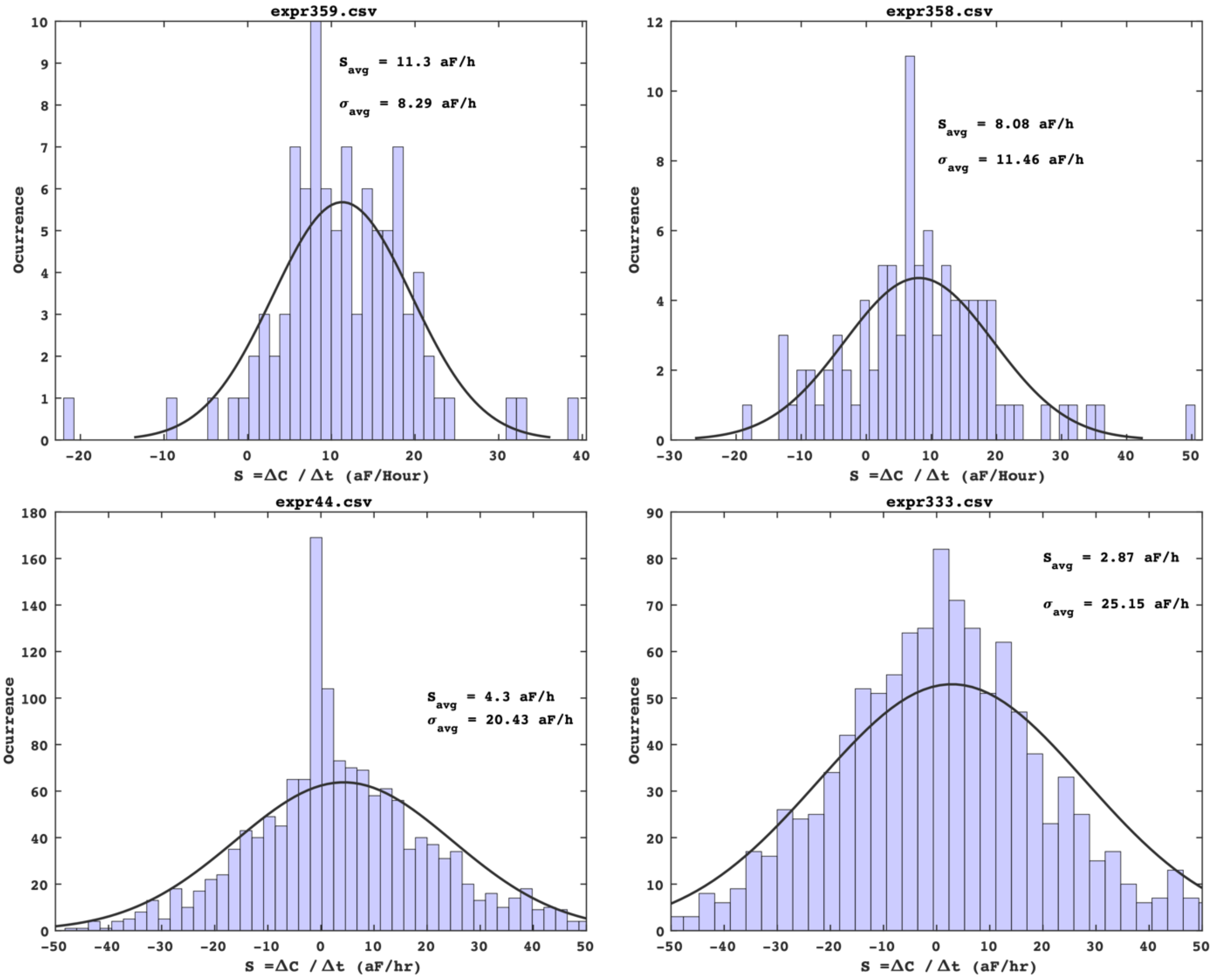

Figure 4. Histograms of four experiments showing average capacitance growth factors across the ROI for a segment length of ~ 5 hours. When the segment length is taken to be 30 hours, the distribution of capacitance growth factors is given by a Gaussian distribution (solid black line).

Once the histogram is constructed and its mean is calculated, we now test for the correlation between the cell numbers in the ROI at various time points and the average capacitance data. The cell numbers are obtained using the microscopy images acquired at various times during the experiment and by using the computer vision code obtained in **cvCode.zip**. These results are loaded into Matlab data structures for further use (e.g., **imgCount358.mat** and **imgCount359.mat**). The correlation charts are then outputted by **meanCap.m**, and they are shown in Figure 5 for the four experiments featured in this document. Furthermore, the **meanCap.m** script implements the model described in Equations (7)-(9) of the main paper. The results of the model are shown in Figure 6.

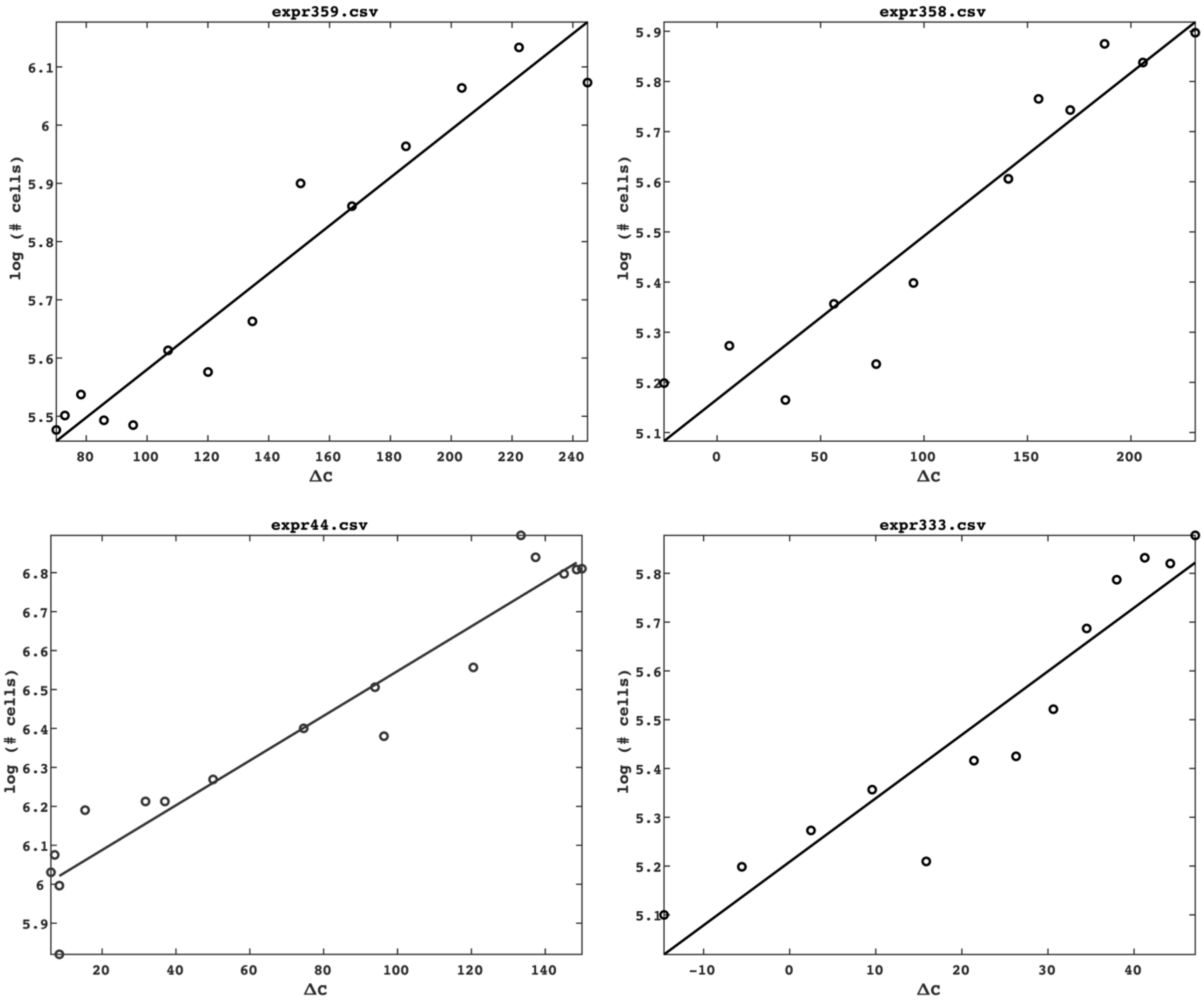

Figure 5. Correlation plots for the four experiments. For each experiment, the cell numbers are outputted by the computer vision code using a series of time-stamped micrographs as its input.

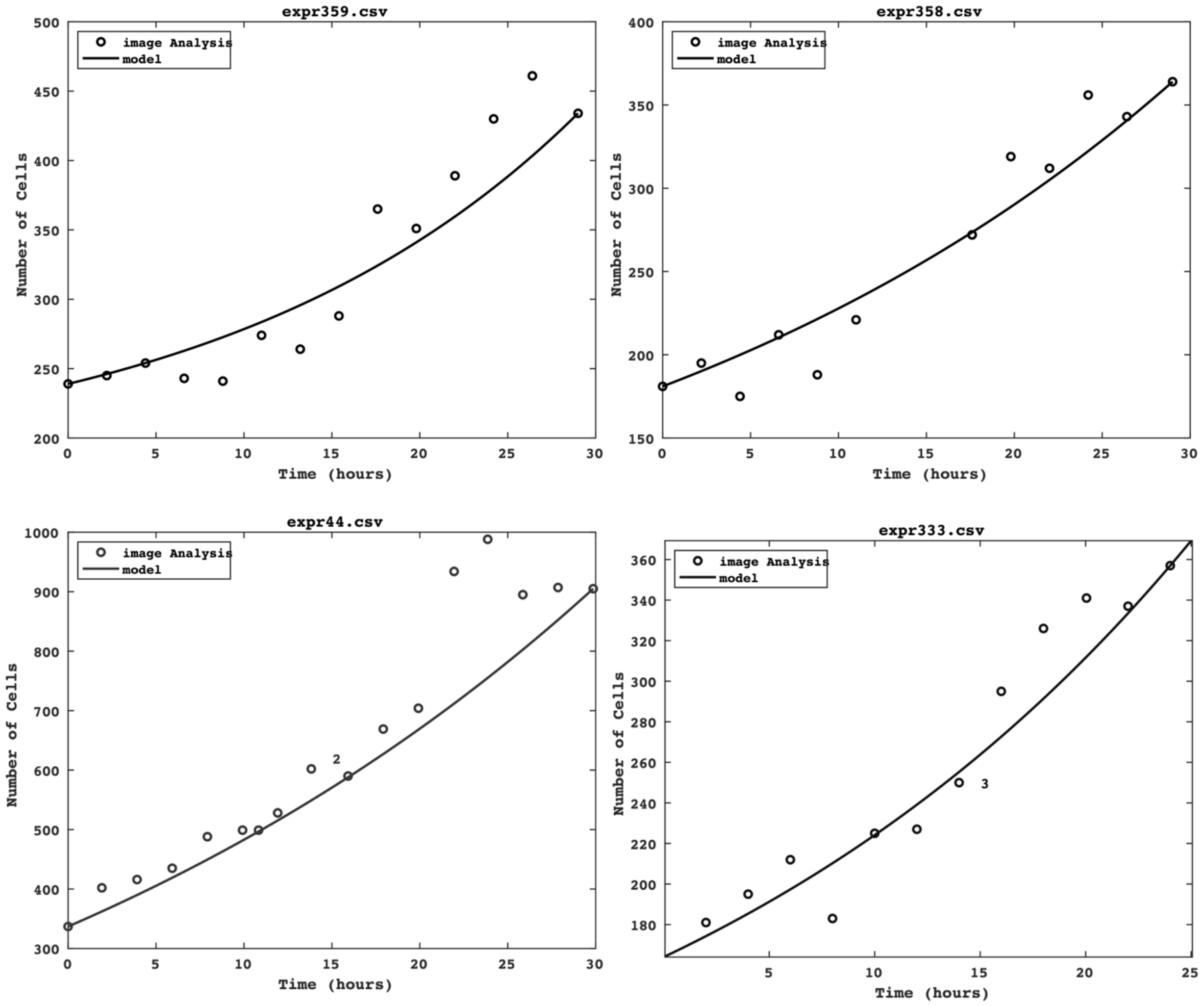

Figure 6. Results of our temporal model for each of the four experiments. For each experiment, the cell numbers are outputted by the computer vision code taking as its input a set of time-stamped micrographs.

2.2.2 Computer Vision Processing

Our computer vision algorithm was written in Python, and it may be found in **cvCode.zip**. The archive includes a "readme" file providing the steps to run the algorithm. Our code was run on a Unix platform, and a sample image with which it can be evaluated is provided in the archive.

The cell detection procedure consists of first post-processing the acquired images to remove non-idealities. For instance, since our culture system is not closed and no active perfusion is used, the cell culture experiences some degree of medium evaporation during cell culture, after some time, despite our two-well mitigation strategy. This alters the optical path and results in a change in focus, eventually leading to degraded image quality. Thus, image pre-processing steps can help remove the blur that results from the change in focus from one time point to another. These effects can be mitigated using a rolling-ball filter (Sternberg, 1983), which acts as a low-pass filter. Here the low-pass operation is designed as a combination of a 2D median filter followed by a downsampling operation. The result of post-processing on a single image is shown in Figure 7, for a worst-case scenario when image quality has degraded. For visualization purposes, the resulting image's intensity histogram is reshaped, and

each grayscale pixel is transformed through a viridis color map, yielding a false color green hue or gray hue.

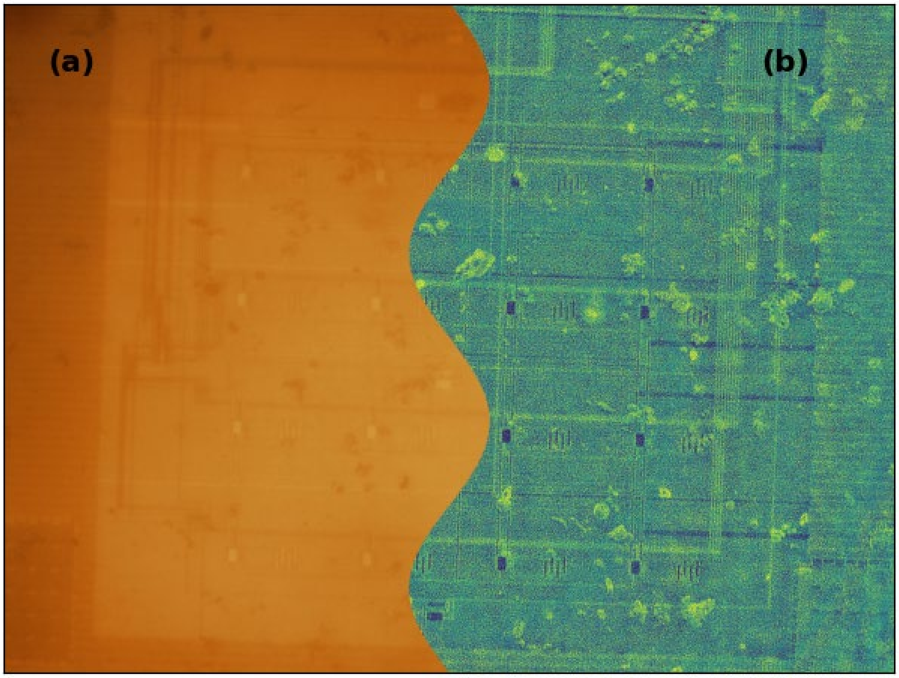

Figure 7. (A) Blurred microscopy image due to degraded focus. (B). Post-processed image where cells and chip features are clearly visible.

Once the image is post-processed, our computer vision executes a correlation filter-based object detection process in which the image is correlated on a pixel-by-pixel basis with a selected template (Bolme, Draper and Beveridge, 2009; Ulman *et al.*, 2017; Kutlu, Avci and Özyurt, 2020). This correlation output, also called correlation image, shows several intensity peaks. These intensity peaks suggest a high likelihood that the region centered at the peak location and the template are similar (Kim and de Araújo, 2007; Ahmad *et al.*, 2018). Such a high likelihood may indicate a feature of interest, such as a cell. The template is constructed manually by using a reference image. A plurality of single cell images is selected from the reference image and overlayed with their centers aligned. The result is a composite image that tells the algorithm what typical cell features look like.

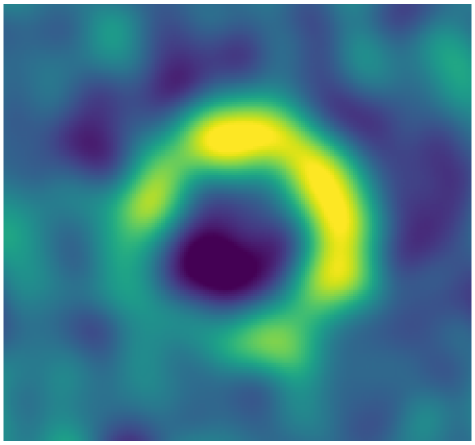

Figure 8. Composite image representing a template that is used by the correlation filter.

Given the differences in cell shapes and background image features (i.e., features of the chip itself), a distribution of correlations is produced by the algorithm. A threshold was used to decide what correlation level was sufficient to conclude that a cell was present at a particular location. Figure 9 shows the cell counts obtained using this method, and Figure 10 shows how we adjusted the threshold as a function of time. The blurrier the image, the lower the threshold. Figure 11 shows the output of the algorithm, which consists of the original image marked with indicators (red squares) where the correlation is found to be highest. Based on the threshold adjustment, we find an average discrepancy of ~10% between the number of cells reported by the algorithm and the actual number of cells obtained by manually counting the cells.

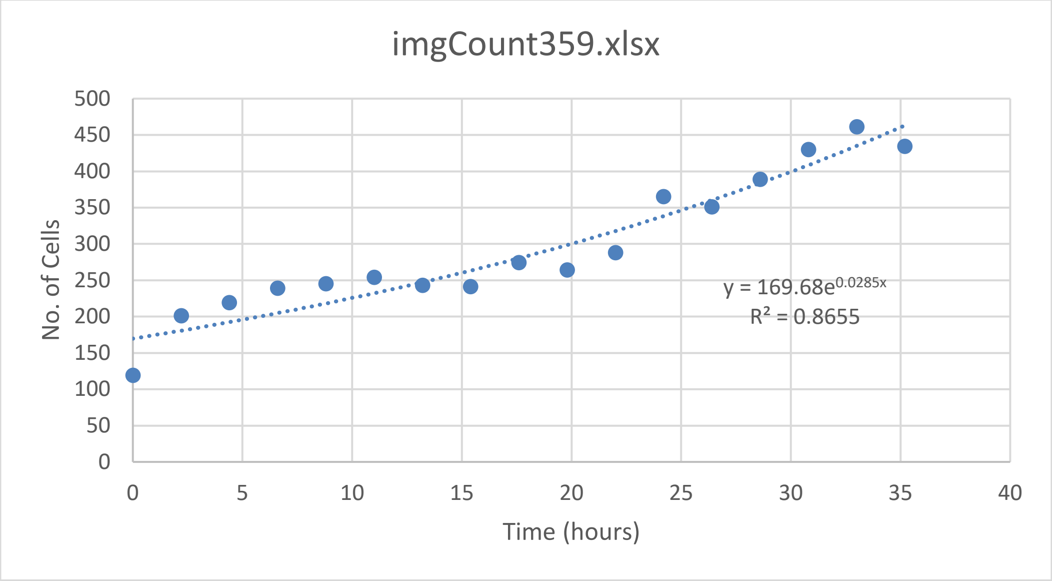

Figure 9. Cell count returned by the algorithm for expr359.csv. The data agree well with an exponential fit and the manual count of cells from each image.

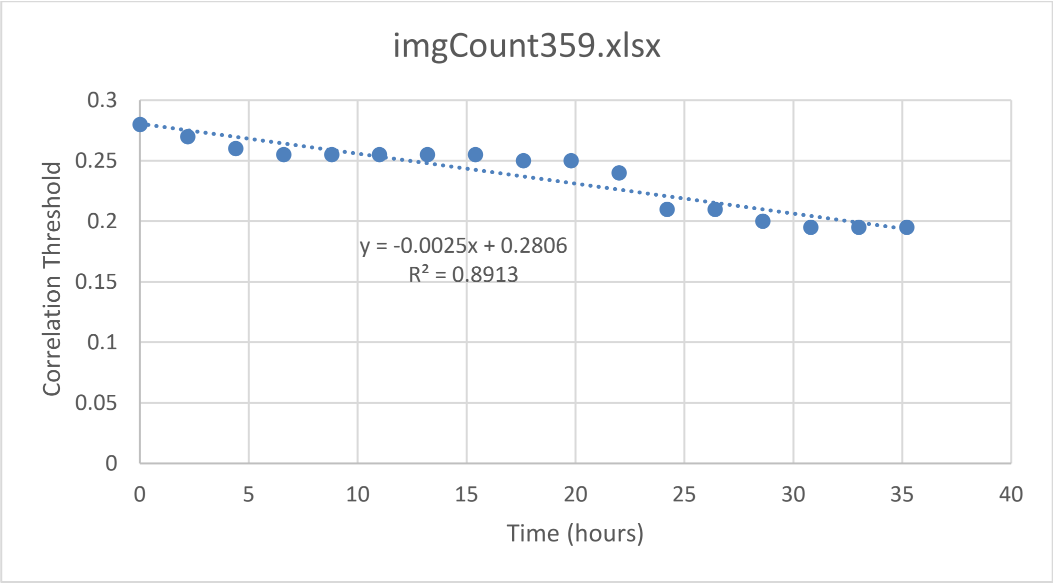

Figure 10. Correlation threshold adjustment as a function time.

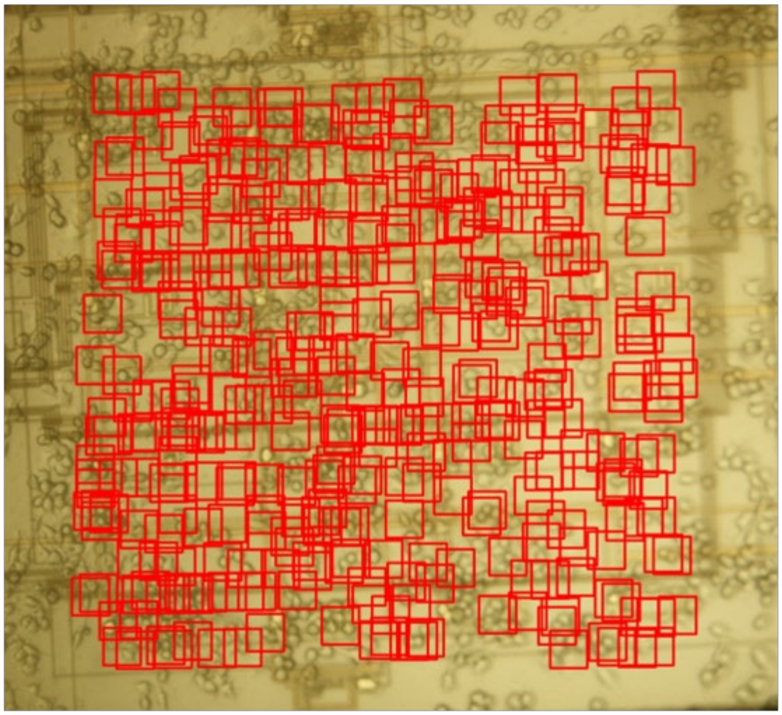

Figure 11. Markers identifying cells detected by the algorithm in the ROI. Note that the algorithm is also provided the x and y coordinates of the ROI, such that it only looks in the ROI for correlations.

2.3 Monitoring Cell Activity

As shown in Figure 1, cell activity was monitored using an upright bright field microscope. In our online repositories linked above, we provide a time-lapse video showing that the cells are dividing inside the ROI. The video confirms that the measured capacitance is a result of the cell population growing and not because of floating cells falling inside the ROI. The video is also available on

YouTube[4].

Snapshots from the **expr359.mp4** video are shown in Figure 12. Two photos of the ROI are shown at different phases of the experiment and confirming that cell divisions are happening inside the ROI. The reader is encouraged to visualize the video to further confirm that the culture is indeed growing in number over time, which means that the measured capacitance is not the result of floating cells adsorbing onto the surface.

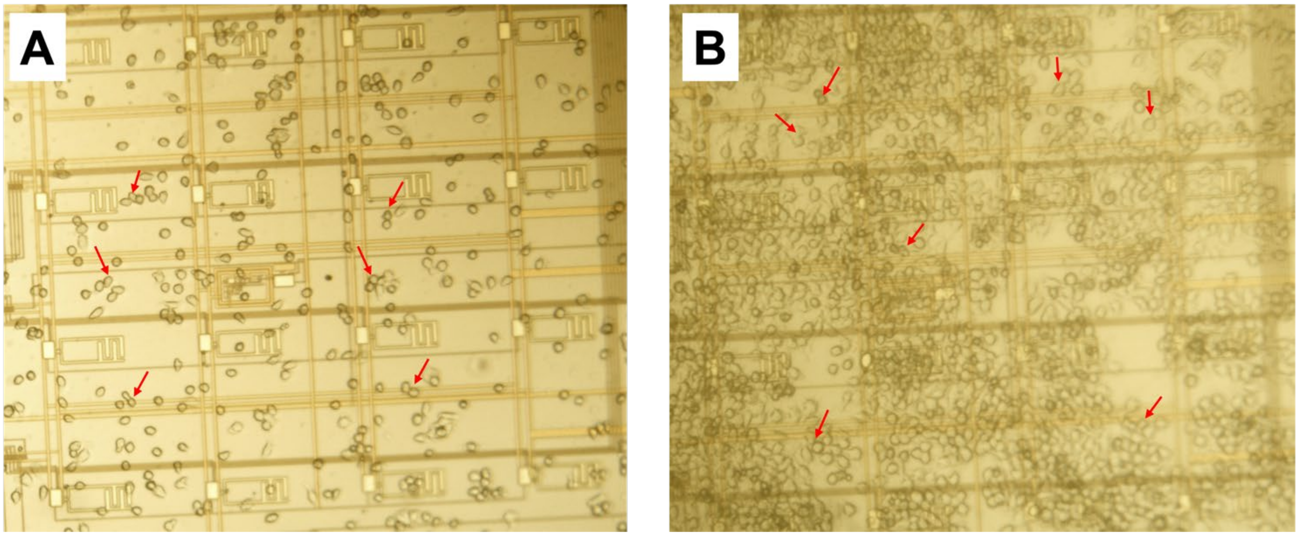

Figure 12. (A) Snapshot from the expr359.mp4 video showing cells inside the ROI at an early phase of the experiment. The red arrows indicate cell division events, which confirms that the number of cells is increasing. (B) Snapshot from the expr359.mp4 video at a later phase of the culture showing a net increase in the number of cells inside the ROI. Cell division events are also marked by red arrows. Notice the blurring incurred relative to the image in panel (A).

To further verify that the conditions were suitable for cell culture growth, we conducted an experiment with a medium that contained Phenol Red, a pH indicator. The pH indicator had a red color, imparting the same color to the cell media at the beginning of the experiment. As can be seen in Figure 13, the medium remained the same color, indicating its pH did not change during the experiment.

[4] https://www.youtube.com/watch?v=pI3L52g69AE

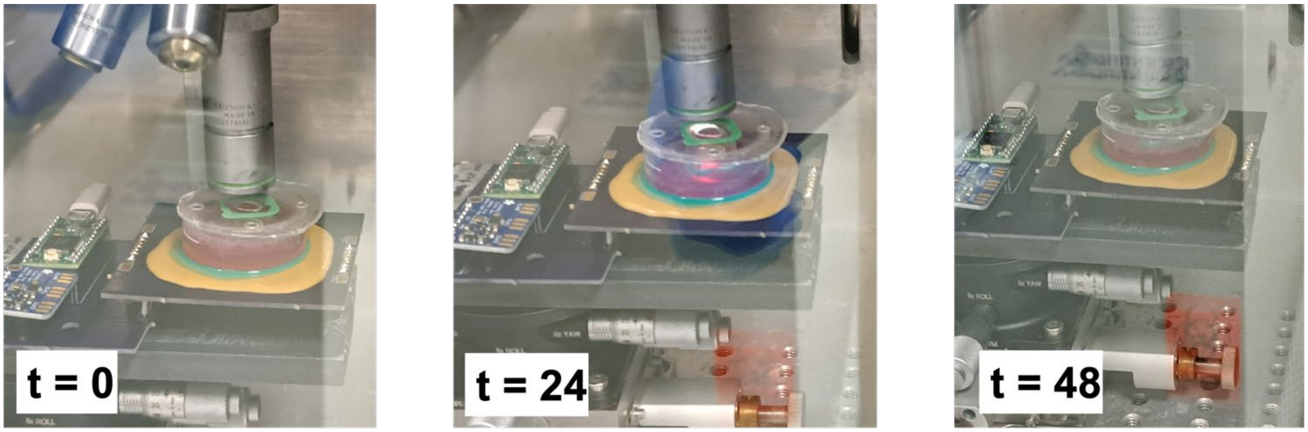

Figure 13. Experiment conducted with a pH indicator (Phenol Red) in the medium. As can be seen from the pictures, the medium retains its color after 48 hours.

Lastly, to further confirm that the measured capacitance responses were due the cells proliferating in the ROI, we conducted an experiment with the medium only. Specifically, we pipetted the same volume of medium as that used in the cell proliferation experiments, but this time, we did not plate cells onto the chip. The results of this experiment are shown in Figure 14 below, which was also obtained by running **meanCap.m**. The drift in signal observed is due to the slow rate evaporation experienced during the experiment, even when using the two-well system. This drift is much smaller and opposite in sign than the overall capacitance measures observed when cells are proliferating in the ROI. This suggests that the measured signal is indeed observed in response to increase cell numbers in the ROI.

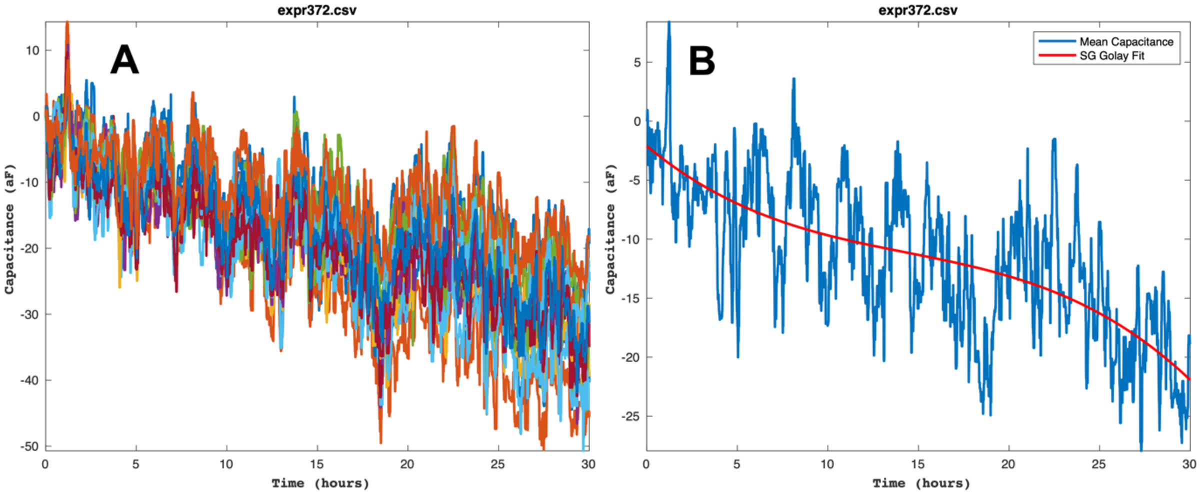

Figure 14. Medium only experiment. (A) Traces for the 16 electrodes. (B) Average measured capacitance and its corresponding SG fit.

2.4 Cost Estimate

To give the reader an idea of the upfront costs we incurred in fabricating the prototype, we have estimated the cost of the various materials and services that were used. Specifically, we estimate the per unit cost of the printed circuit board, wire bonding services, the cost of a single unit of the integrated circuit chip, and the cost of the epoxy encapsulation per unit. The cost of the culture chamber materials was not accounted in this estimate because it was negligible. In total, a single unit of our prototype

was fabricated for less than $20.00. References are provided for each item and service that were used in order to provide the reader an overall view of the market at the time of writing this document.

Table 2. Cost estimate for the prototype used in this study.

Item/Service	Estimated Cost/unit	Reference
**PCB**	$ 10.00	https://blog.svtronics.com/how-much-does-a-pcb-cost-factors-that-will-affect-the-price/
**Wirebonding**	$ 5.00	https://ieeexplore.ieee.org/document/5490877
**Capacitance sensing IC**	$ 0.35	https://electronics-sourcing.com/2018/07/17/rising-demand-from-multiple-segments-drive-analog-ic-market-by-james-carbone/
**Epoxy**	$ 0.86	Based on a quote received from Cotronics on 3/21/2023 and on the amount used to encapsulate the wirebonds and make the supporting platform.
**Total**	$ 16.21	

2.5 Supporting Files

Below, we provide a list of supporting documents and files in the digital archives linked above. They include the measurement files outputted in **.csv** by the code controlling the chip, m-files in **.m** format used for time series data processing, Python files for cell counting. We further provide a sample video of a representative experiment. Table 3 below summarizes the supporting documents that are provided.

Table 3. List of the files provided with this document.

Filename	Type	Description
**meanCap.m**	Matlab	m-file
**slopeCompute.m**	Matlab	m-file
**expr359.mp4**	VLC Media Player	30-hr sped-up time-lapse video
**expr358.csv**	MS Excel	Measurement data file
**expr359.csv**	MS Excel	Measurement data file
**expr372.csv**	MS Excel	Measurement data file

**imgCount359.mat**	Matlab	Cell numbers **.mat** data structure for **expr359.csv**
**imgCount358.mat**	Matlab	Cell numbers **.mat** data structure for **expr358.csv**
**cvCode.zip**	WinZip to unzip and Python to run;	Computer vision algorithm: please use **readme.txt** file in the archive along with sample figures also located therein

3 References

Ahmad, A. *et al.* (2018) 'Correlation Filters for Detection of Cellular Nuclei in Histopathology Images', *Journal of Medical Systems*, 42(1), p. 7. doi:10.1007/s10916-017-0863-8.

Bolme, D.S., Draper, B.A. and Beveridge, J.R. (2009) 'Average of Synthetic Exact Filters', in *2009 IEEE Conference on Computer Vision and Pattern Recognition*. IEEE, pp. 2105–2112. doi:10.1109/CVPR.2009.5206701.

Gilpin, Y. *et al.* (2022) 'Tracking the Effects of Tumor Treating Fields on Human Breast Cancer Cells in vitro Using a Capacitance Sensing Lab-on-CMOS Microsystem', in *2022 29th IEEE International Conference on Electronics, Circuits and Systems (ICECS)*. IEEE, pp. 1–4. doi:10.1109/ICECS202256217.2022.9971093.

Kim, H.Y. and de Araújo, S.A. (2007) 'Grayscale Template-Matching Invariant to Rotation, Scale, Translation, Brightness and Contrast', in, pp. 100–113. doi:10.1007/978-3-540-77129-6_13.

Kutlu, H., Avci, E. and Özyurt, F. (2020) 'White blood cells detection and classification based on regional convolutional neural networks', *Medical Hypotheses*, 135, p. 109472. doi:10.1016/j.mehy.2019.109472.

Senevirathna, B. *et al.* (2019) 'An Imaging Platform for Real-Time In Vitro Microscopic Imaging for Lab-on-CMOS Systems', in *2019 IEEE Biomedical Circuits and Systems Conference (BioCAS)*. IEEE, pp. 1–4. doi:10.1109/BIOCAS.2019.8919023.

Sternberg (1983) 'Biomedical Image Processing', *Computer*, 16(1), pp. 22–34. doi:10.1109/MC.1983.1654163.

Ulman, V. *et al.* (2017) 'An objective comparison of cell-tracking algorithms', *Nature Methods*, 14(12), pp. 1141–1152. doi:10.1038/nmeth.4473.
